# The impact of comorbidities on post-operative complications following colorectal cancer surgery

**DOI:** 10.1371/journal.pone.0243995

**Published:** 2020-12-23

**Authors:** David E. Flynn, Derek Mao, Stephanie T. Yerkovich, Robert Franz, Harish Iswariah, Andrew Hughes, Ian M. Shaw, Diana P. L. Tam, Manju D. Chandrasegaram

**Affiliations:** 1 Department of General Surgery, The Prince Charles Hospital, Brisbane, Queensland, Australia; 2 The Common Good Foundation, The Prince Charles Hospital, Brisbane, Queensland, Australia; Cleveland Clinic, UNITED STATES

## Abstract

**Background:**

Colorectal cancer surgery is complex and can result in severe post-operative complications. Optimisation of surgical outcomes requires a thorough understanding of the background complexity and comorbid status of patients.

**Aim:**

The aim of this study is to determine whether certain pre-existing comorbidities are associated with high grade post-operative complications following colorectal cancer surgery. The study also aims to define the prevalence of demographic, comorbid and surgical features in a population undergoing colorectal cancer resection.

**Method:**

A colorectal cancer database at The Prince Charles Hospital was established to capture detailed information on patient background, comorbidities and clinicopathological features. A single-centre retrospective study was undertaken to assess the effect of comorbidities on post-operative outcomes following colorectal cancer resection. Five hundred and thirty-three patients were reviewed between 2010–2018 to assess if specific comorbidities were associated with higher grade post-operative complications. A Clavien-Dindo grade of three or higher was defined as a high grade complication.

**Results:**

Fifty-eight percent of all patients had an ASA grade of ASA III or above. The average BMI of patients undergoing resection was 28 ± 6.0. Sixteen percent of all patients experienced a high grade complications. Patients with high grade complications had a higher mean average age compared to patients with low grade or no post-operative complications (74 years vs 70 years, p = 0.01). Univariate analysis revealed patients with atrial fibrillation, COPD, ischaemic heart disease and heart failure had an increased risk of high grade complications. Multivariate analysis revealed pre-existing atrial fibrillation (OR 2.70, 95% CI 1.53–4.89, p <0.01) and COPD (OR 2.02 1.07–3.80, p = 0.029) were independently associated with an increased risk of high grade complications.

**Conclusion:**

Pre-existing atrial fibrillation and COPD are independent risk factors for high grade complications. Targeted perioperative management is necessary to optimise outcomes.

## Introduction

Colorectal cancer (CRC) is the third most common cancer in Australia, with over 17 000 people diagnosed annually [1]. Surgical resection of colorectal cancer is the cornerstone of treatment, however colorectal surgery can be complex. National morbidity rates for colorectal cancer resection are benchmarked at 21% with an in-patient mortality rate of 1.1% [2]. While complications are a known entity with colorectal surgery, there is an increasing need to examine our patients in order to optimise surgical outcomes [2–4]. This is especially relevant given the aging and highly comorbid population who present with colorectal cancer. Within the surgical community, there is a general consensus that comorbidities play an important role in surgical outcomes with age, cardiovascular disease and diabetes having already been shown to increase post-operative morbidity [3–5].

Current larger scale colorectal databases rarely capture granular details surrounding patient comorbidities. This can make it difficult to ascertain which specific comorbidities have the greatest negative impact on post-surgical outcomes. The Charlson Comorbidity Index has widely been used to evaluate comorbid burden as a measured index to predict mortality. However, this classification system uses grouped comorbidity groups for analysis, thereby lacking disease-specific data and preventing analysis on the specific impact of certain diseases [6–8].

As well as comorbidity analysis, the assessment of post-operative outcomes has also varied widely in literature and within colorectal cancer databases. Several factors can influence the incidence of complications. The definition of ‘post-operative complications’ and the period which is defined as ‘post-operative’ are examples of the variation in complication monitoring. The Clavien-Dindo Classifications are an objective, reproducible and standardised categorisation system, developed for comparing post-operative complications. The classifications have been validated for use in several surgical subspecialties include hepatobiliary and urological surgery [9, 10].

While there is literature that outlines the prevalence of comorbidities in certain tumour types, there is a paucity of information on colorectal cancer. Moreover, there is a lack of consistency in grading and comparing postoperative outcomes within these studies.

The few studies that observe the effect of comorbidities on post-operative outcomes were published over ten years ago and reflect older surgical techniques, standards and treatment guidelines [11–13].

The aims of this study are to 1) Determine the prevalence of comorbidities, surgical and pathological features of patients with colorectal cancer and; 2) Describe the association between specific patient comorbidities and high grade post-operative complications following colorectal cancer surgery.

## Materials and methods

### Study design and data source

The Prince Charles Hospital (TPCH) in Brisbane, Australia is a tertiary cardiothoracic centre that cares for patients with advanced and complex heart and lung diseases. The General Surgical department at TPCH provides care for colorectal cancer patients whose clinical background and comorbidities are captured in a detailed clinicopathological database. A retrospective study using The Prince Charles Hospital colorectal cancer database was undertaken to investigate the effect of patient comorbidities on post-operative outcomes. Patient charts were individually reviewed and data extracted by trained medical personnel. Data was obtained from previous and current admissions as well as correspondence letters, follow up documentation and outpatient/readmission notes.

### Study population

Patients who underwent surgical resection of colorectal cancer at The Prince Charles Hospital between January 2010 and December 2018 were included in the study.

Patients were excluded if they had endoscopic resection of the malignant lesion without surgical intervention. Patients below the age of 12 were also excluded from the study.

### Measures and definitions

Basic demographic data such as age, gender, date of birth, height, weight, body mass index (BMI) and American Society of Anaesthesiologists (ASA) grade were documented. Specific pre-operative conditions were grouped into comorbid categories: cardiovascular, respiratory, metabolic, autoimmune and renal disease.

### Cardiovascular comorbidities

Cardiovascular diseases recorded included ischemic heart disease, previous coronary artery bypass grafting, previous percutaneous coronary intervention, pacemaker insertion, defibrillator insertion (AICD), previous valve repair, heart failure (any type), cardiomyopathy, hypertension, pulmonary hypertension and atrial fibrillation.

### Respiratory comorbidities

Respiratory diseases recorded included asthma, COPD, bronchiectasis, cystic fibrosis and obstructive sleep apnoea.

### Metabolic, autoimmune and renal disorders

Metabolic diseases recorded included type I diabetes, type II diabetes and hyperlipidaemia. Autoimmune conditions recorded included rheumatoid arthritis, psoriasis, polymyalgia rheumatica, and systemic lupus erythematosus (SLE). Baseline renal function was noted in each patient.

### Surgical and pathological features

Surgical data included the operation performed, urgency of surgery, operative approach, discharge date and length of stay. Pathological data included tumour histopathology, histological grade and TNM stage of disease.

### Post-operative outcomes

The database and patient charts were reviewed for specific post-operative complications and their treatment was documented. Complications were Graded from I to V according to the Clavien-Dindo Classification of Surgical Complications (S1 Appendix). Complication grades were divided into low grade complications (Clavien-Dindo grades I-II) and high grade complications (Clavien-Dindo grades III-V) for analysis. Post-operative complications were defined as those that arose up to 60 days post-operatively. Grading was performed by trained medical clinicians. Quality assurance of collected data was undertaken by an independently trained clinician.

### Statistical analysis

Differences in demographic features, comorbidities and surgical/pathological features between the two complication groups were assessed using t-tests and Fisher’s exact tests as appropriate. Statistically significant results were defined as those with p ≤ 0.05.

The association between comorbidities and high grade post-operative complications was assessed using uni- and multivariate cox regressional analysis to assess odds ratios for each comorbidity. All comorbidities were assessed using univariate analysis, while only those that were found to be statistically significant were analysed in the multivariate model. Statistical review was performed by a qualified biomedical statistician. Data was analysed with Stata v14 software (StataCorp).

### Ethics

Ethics approval for this database was granted by the Prince Charles Hospital Human Research Ethics Committee (Reference: HREC/17/QPCH/295). A waiver of consent was approved to allow access to confidential patient information without consent. Patients were not anonymized prior to data collection. However, patient names and certain other identifying data was not recorded in the database to help guard against confidentiality breaches. Patient data was accessed between January 2018 and June 2019.

## Results

Five hundred and thirty-three patients who underwent colorectal cancer resection between January 2010 and December 2018 were reviewed.

### Demographics

The mean age of patients undergoing colorectal cancer surgery was 67.4 years ± 15.6 SD. The mean BMI was 27 ± 6.0 SD. There was a 50:50 even distribution of males and females within the cohort and the majority of patients (n- = 309, 58%) had an ASA of ≥3 (indicating the patient is living with a severe disease process).

### Comorbidities

Fifty-nine percent of all patients who underwent colorectal cancer resection had a cardiac comorbidity, with hypertension (n = 266, 50%) being the most common condition followed by ischemic heart disease (n = 98, 18%) and atrial fibrillation (n = 71, 13%).

Twenty-eight percent of patients had respiratory disease with asthma (n = 58, 11%) and COPD (n = 62, 12%) being the most common lung conditions. Fifty-four percent of all patients had metabolic disease (n = 288) with hyperlipidaemia being the most common (n = 158, 30%) followed by type II diabetes (n = 96, 18%). Only 2% of patients (n = 11) had an autoimmune disease. The majority of patients had a preoperative estimated glomerular filtration rate (eGFR) >90 ml/min/1.73m2 (n = 304, 57%) which is indicative of no underlying renal disease. Thirty percent of the cohort had an eGFR between 60–89 while 9% had an eGFR between 45–59. Forty-three percent of patients had a degree of renal impairment prior to surgery. Fourteen percent of patients were current smokers at the time of surgery (n = 76). Table 1 outlines the demographic data and pre-morbid features of the patients.

**Table 1:** Demographics and comorbidities of patients who underwent colorectal cancer resections at The Prince Charles Hospital between 2010–2018.

Feature	Number of nil/ low grade complications	Number of high grade complications	Total	*P*-value
	(% of nil/low grade complications)	(% of high grade complications)		
Patients	448 (84)	85 (16)	533	
Gender				
• Male	215 (48)	52 (61)	267	0.03
• Female	233 (52)	33 (39)	266	
Median age	70	74	67	0.01
• (range)	17–96	31–90	17–96	
Median BMI	27	28	27	
• (range)	15–59	15–40		
• <20	31 (7)	6 (7)	37	0.24
• 20–24.9	119 (27)	21 (25)	140	
• 25–29.9	171 (38)	27 (32)	198	
• 30–39.9	110 (25)	30 (35)	140	
• >40	17 (4)	1 (1)	18	
Median ASA	3	3		
• Grade I	37 (8)	4 (5)	41	0.016
• Grade II	165 (37)	19 (22)	185	
• Grade III	211 (47)	49 (58)	259	
• Grade IV	34 (8)	13 (15)	47	
• Grade V	1 (0)	0 (0)	1	
Cardiac				
** •** Ischaemic Heart Disease	74 (17)	24 (28)	98	0.014
** •** Coronary Artery Bypass Graft	29 (7)	11 (13)	40	0.045
** •** Percutaneous Coronary Intervention	26 (6)	7 (8)	33	0.46
** •** Pacemaker insertion	8 (2)	1 (1)	9	0.69
** •** Cardiac valve replacement	17 (4)	3 (4)	20	1.0
** •** Heart failure (all types)	14 (3)	8 (9)	22	0.014
** •** Cardiomyopathy	10 (2)	8 (9)	18	<0.001
** •** AICD insertion	6 (1)	1 (1)	7	0.90
** •** Hypertension	224 (50)	42 (50)	266	1.0
** •** Pulmonary hypertension	5 (1)	1 (1)	6	0.903
** •** Atrial fibrillation	48 (10)	23 (27)	71	<0.001
Respiratory				
** •** Asthma	49 (11)	9 (11)	58	1.0
** •** COPD	44 (9)	18 (21)	62	0.005
** •** Bronchiectasis	6 (1)	0 (0)	6	-
** •** Cystic fibrosis	1 (0)	0 (0)	1	-
** •** Obstructive sleep apnoea	28 (6)	6 (7)	34	0.81
Metabolic				
** •** Type I Diabetes	2 (0)	0 (0)	2	-
** •** Type II Diabetes	84 (19)	12 (14)	96	0.36
** •** Hyperlipidaemia	131 (29)	27 (32)	158	0.70
Previous cancer	59 (13)	17 (20)	76	0.098
Autoimmune				
** •** Rheumatoid arthritis	3 (1)	0 (0)	3	0.80
** •** Psoraisis	8 (2)	1 (1)	9	
** •** Polymyalgia rheumatica	6 (1)	0 (0)	6	
** •** SLE	1 (0)	0 (0)	1	
Pre-operative renal function				
** •** eGFR >90	260 (58)	44 (52)	304	0.60
** •** eGFR 60–89	133 (30)	25 (30)	158	
** •** eGFR 45–59	38 (9)	11 (13)	49	
** •** eGFR 30–44	16 (4)	3 (4)	19	
** •** eGFR 15–29	2 (1)	1 (1)	3	
** •** eGFR < 15	0 (0)	0 (0)	0	
Current Smoker (within 12 months)	63 (14)	13 (15)	76	

BMI, Body Mass Index; ASA, American Society of Anaesthesiologists Physical Status Classification; AICD, Automatic Implantable Cardioverter Defibrillator; COPD, Chronic Obstructive Pulmonary Disease; SLE, Systemic lupus erythematosus.

### Surgical and pathological features

The majority of cancers (n = 304, 57%) were located within the right side of the colon (from the caecum to the hepatic flexure). Left sided colon cancers (from the splenic flexure to the sigmoid) made up 30% of cases (n = 161) while rectal cancers made up 17% (n = 88).

Right hemicolectomies, extended right hemicolectomies, appendicectomies and caecectomies (n = 294, 55%) made up the majority of surgical cases and reflects the dominance of right sided colon cancers. The majority of surgical cases were performed laparoscopically (including laparoscopically assisted) (n = 410, 77%) while open cases (including laparoscopic converted to open) made up 23% of all operations (n = 121).

The most common histopathology was adenocarcinoma of no special type (n = 436, 82%). Most patients in both the nil/low and high grade complications groups had an early stage of caner (Stage I and IIA). The majority of our patients had stage I disease, with no difference in distribution of disease between the two groups (p = 0.73). The surgical and pathological features of the cohort are outlined in Table 2.

**Table 2:** Surgical and pathological features of patients who underwent colorectal cancer resections at The Prince Charles Hospital between 2010–2018.

Feature	Number of nil/ low grade complications	Number of high grade complications	Total	*P*-value
	(% of nil/low grade complications)	(% of high grade complications)		
Patients	448 (84)	85 (16)	553	
Median LOS (days)	6	16	10	
** •** < 14 days	398 (89)	36 (42)	434	<0.001
** •** ≥ 14 days	43 (11)	49 (58)	92	
30-day mortality	3 (1)	6 (7)	9	<0.001
Surgical Urgency				
** •** Emergency	64 (14)	19 (22)	83	0.13
** •** Urgent	356 (79)	59 (70)	415	
** •** Elective	27 (6)	6 (7)	33	
Location of cancer				
** •** Caecum to transverse colon	259 (58)	45 (53)	304	0.11
** •** Splenic flexure to sigmoid	137 (31)	24 (28)	161	
** •** Rectum/Anus	67 (15)	21 (25)	88	
Type of operation				
** •** Left hemicolectomy	23 (5)	3 (4)	26	0.25
** •** Right hemicolectomy	182 (41)	34 (40)	216	
** •** Extended right hemicolectomy	36 (8)	11 (13)	47	
** •** Total colectomy	6 (1)	0 (0)	6	
** •** Subtotal colectomy	7 (2)	3 (4)	10	
** •** High anterior resection	82 (18)	12 (14)	94	
** •** Low anterior resection	24 (5)	6 (7)	30	
** •** Ultralow anterior resection	24 (5)	7 (8)	31	
** •** Hartmann’s procedure	18 (4)	4 (5)	22	
** •** Abdominoperineal resection	5 (1)	2 (2)	7	
** •** Appendicectomy	30 (7)	0 (0)	30	
** •** TEMS/TAMIS	2 (0)	0 (0)	2	
** •** Other	9 (2)	3 (4)	12	
Approach				
** •** Laparoscopic	274 (61)	37 (44)	311	0.012
** •** Laparoscopic assisted	82 (18)	17 (20)	99	
** •** Laparoscopic converted to open	31 (7)	11 (13)	42	
** •** Open (including local excision)	59 (13)	20 (24)	79	
** •** TA-TME	2 (0)	0 (0)	2	
Histological diagnosis				
** •** Adenocarcinoma	359 (80)	77 (91)	436	<0.001
** •** Mucinous Adenocarcinoma	54 (12)	5 (6)	59	
** •** Signet Ring Cell Carcinoma	0 (0)	2 (2)	2	
** •** Carcinoid	24 (5)	0 (0)	24	
** •** Other	11 (2)	1 (1)	12	
Histological Grade				
** •** Low Grade	340 (76)	58 (68)	398	0.017
** •** High Grade	84 (19)	26 (31)	110	
** •** No Grade	24 (5)	1 (1)	25	
Stage of Disease				
** •** I	124 (28)	19 (22)	143	0.73
** •** IIA	112 (25)	22 (26)	134	
** •** IIB	31 (7)	5 (6)	36	
** •** IIIA	12 (3)	2 (2)	14	
** •** IIIB	83 (17)	16 (19)	99	
** •** IIIC	33 (7)	11 (13)	44	
** •** IV	53 (11)	10 (12)	63	

LOS, length of stay; TEMS, transanal endoscopic microsurgery; TAMIS, transanal minimally invasive surgery; TA-TME, transanal total mesorectal excision.

### Complications

Complications were divided into surgical complications and medical complications.

One hundred and twenty-nine patients (24%) developed surgical complications.

The most common surgical complications were prolonged ileus (n = 108, 20%), superficial wound infection (n = 40, 8%) and an abdominopelvic collection (n = 19, 4%). Anastomotic leaks accounted for 2.6% of complications (n = 14). One hundred and twenty patients (23%) had a medical complication. The most common medical complications were cardiac arrhythmias (n = 49, 9%), respiratory infections (n = 36, 7%) and impaired renal function (n = 14, 3%). The median length of stay (LOS) for patients following surgery was 10 days with a 30-day mortality rate of 2% (n = 9). The surgical and medical complications of the patients are outlined in Table 3.

**Table 3:** Surgical and pathological features of patients who underwent colorectal cancer resections at The Prince Charles Hospital between 2010–2018.

Complication	Number of nil/ low grade complication	Number of high-grade complications	Total (% of total)
Surgical Complications			
** •** Abdominopelvic collection	4	15	19 (4)
** •** Anastomotic leak	1	14	15 (3)
** •** Enterocutaneous fistula	0	1	1 (1)
** •** Superficial wound dehiscence	3	4	7 (1)
** •** Wound infection	15	25	40 (8)
** •** Prolonged ileus	41	67	108 (2)
** •** Small bowel obstruction	1	7	8 (2)
** •** Urinary retention	5	6	11 (2)
** •** Return to theatre	0	17	17 (3)
** •** Anastomotic leak	0	14	14 (3)
Medical Complications			
** •** Deep vein thrombosis	2	4	6 (1)
** •** Pulmonary embolism	0	1	1 (1)
** •** Respiratory infection	13	23	36 (7)
** •** Ischaemic cardiac event	0	8	8 (2)
** •** Cardiac arrhythmia	18	31	49 (9)
** •** Cerebrovascular event	2	2	4 (1)
** •** Respiratory failure	0	17	17 (3)
** •** Renal failure	0	14	14 (3)

### Patient background and comorbidities associated with high grade complications

Patients who had Clavien-Dindo Complication Classifications graded III-V were classified as having high grade post-operative complications. The demographic features of high-grade post-operative complications are outlined in Table 1.

Out of the entire study cohort, 85 patients (16%) had high grade post-operative complications. Patients who had high grade post-operative complications were older than those with nil/low grade complications (median 74 vs 70 years old, p = 0.01).

Those with high grade complications had a higher proportion of patients with ASA III-V when compared to those with nil/low grade complications (73% vs 55%, p = 0.016).

Patients with high grade complications were found to have higher rates of ischemic heart disease (28% vs 17%, p = 0.014), coronary artery bypass grafting (13 vs 7, p = 0.045), heart failure (9% vs 3%, p = 0.014), cardiomyopathy (9% vs 2%, p<0.001) and atrial fibrillation (27% vs 10%, p = <0.001).

Patients with high grade complications were also found to have double the incidence of COPD (21% vs 9%, p = 0.005) (Table 1). There was no significant difference between the two groups with regards the incidence of type II diabetes, hyperlipidaemia and baseline renal function.

### Univariate analysis of comorbidities associated with high grade complications

Patient background data, comorbidities and their association with high grade complications was analysed by univariate analysis and presented in Table 4. The risk of complications was assessed for every 5 years of life and for every 5 BMI units.

**Table 4:** Univariate analysis of comorbidities and their association with high grade post-operative complications.

Feature	Number of high grade complications	Unadjusted Odds Ratio (95% Confidence Intervals)	*P*-value
	(% of high grade complications)		
Atrial fibrillation	23 (27)	3.09 (1.76, 5.43)	<0.001
Age (5 years)	85 (100)	1.02 (1.00, 1.04)	0.008
COPD	18 (21)	2.47 (1.35, 4.52)	0.005
Ischaemic Heart Disease	24 (28)	1.99 (1.17, 3.39)	0.012
Heart Failure (all types)	8 (9)	3.22 (1.31, 7.94)	0.017
Coronary Artery Bypass Graft	11 (13)	2.14 (1.02, 4.49)	0.053
Type II Diabetes	12 (14)	0.71 (0.37, 1.37)	0.30
Obstructive sleep apnoea	6 (7)	1.14 (0.46, 2.84)	0.78
Hyperlipidaemia	27 (32)	1.12 (0.68, 1.86)	0.64
Asthma	9 (11)	0.96 (0.46, 2.05)	0.92
Percutaneous Coronary Intervention	7 (8)	1.45 (0.61, 3.47)	0.411
Hypertension	42 (50)	0.98 (0.61, 1.55)	0.92
BMI (5 units)	85 (100)	1.04 (0.85, 1.25)	0.70
ASA			
•Grade II	19 (22)	1.06 (0.34, 3.32)	0.90
•Grade III	49 (58)	2.14 (0.73, 6.31)	0.16
•Grade IV	13 (15)	3.54 (1.05, 11.90)	0.041

Age (5 years), the risk of high grade complications was analysed per every 5 years of life; BMI (5 units), the risk of high grade complications was analsyed per every 5 units of BMI.

Patients with pre-existing atrial fibrillation (OR 3.09, 95% CI 1.76–5.43, p< 0.001), heart failure (OR 3.22, 95% CI 1.31–7.94, p = 0.017) and an ASA IV grade (OR 3.54, 95% CI 1.05–11.90, p = 0.04) had a threefold higher risk of high grade complications when compared to patients who did not have these comorbidities. Patients with chronic obstructive pulmonary disease (OR 2.47, 95% CI 1.35–4.52, p = 0.05) and ischaemic heart disease (OR 1.99, 95% CI 1.17–3.39, p = 0.012) had approximately double the risk of developing high grade complications (Table 4).

Coronary artery bypass grafting was found to be just above the threshold for statistical significance (OR 2.14, 95% CI 1.02–4.49, p = 0.053). Types II diabetes, obstructive sleep apnoea, hyperlipidaemia, asthma and percutaneous coronary intervention were not found to be associated with high grade complications.

Multivariable logistic analysis (Table 5) was performed to allow for adjustment for relevant factors. Only comorbidities that were statistically significant in univariate analysis were analysed with multivariate analysis.

**Table 5:** Factors associated with high grade post-operative complications with multi-regression analysis.

Feature	Adjusted OR (95% CI)	P-value
Atrial fibrillation	2.70 (1.53, 4.89)	<0.001
COPD	2.02 (1.07, 3.80)	0.029
Age (5 years)	1.01 (0.99, 1.03)	0.061
Heart Failure	2.06 (0.79, 5.36)	0.14
CABG	1.54 (0.71, 3.35)	0.27

Of the six comorbidities found to be associated with high grade complications, pre-existing atrial fibrillation (OR 2.70, 95% CI 1.53–4.89, p = 0.001) and COPD (OR 2.02, 95% CI 1.07–3.80, p = 0.029) were the only comorbidities independently associated with high grade post-operative complications.

## Discussion

We performed a retrospective study to determine the effect of comorbidities on surgical outcomes following colorectal cancer resection.

Out study demonstrated that patients with pre-existing atrial fibrillation were almost three times more likely to have severe post-operative complications while patients with COPD were found to be twice as likely to have similar complications.

The independent association between atrial fibrillation and poorer surgical outcomes has never before been described in literature.

Our results demonstrate that patients with high grade complications following colorectal cancer surgery were more likely to be older, have significantly higher rates of ischaemic heart disease, previous coronary artery bypass grafting, atrial fibrillation, heart failure and COPD. This is of particular interest as previous studies have associated ‘cardiac disease’ with post-operative complications without delineating the effect of specific comorbidities. While ischaemic heart disease, heart failure and increased age were found to be associated with the development of high-grade complications on univariate regression analysis they were not independently associated with high grade complications on multivariate analysis.

Atrial fibrillation (AF) is a common cardiovascular comorbidity that frequently occurs within a similar demographic to that of CRC. Approximately 5% of Australia’s population over the age of fifty-five have atrial fibrillation [14]. Although common, atrial fibrillation is by no means innocuous as it predisposes patients to thromboembolism, haemodynamic compromise and arrhythmogenesis [15]. Associated issues regarding ventricular rate control and anti-coagulation in the peri-operative setting can further predispose patients with AF to haemodynamic compromise and post-operative bleeding [16, 17].

Several studies have demonstrated an association between cardiovascular disease and an increase in post-operative complications [18, 19], however, none of these studies have delineated the patient’s specific type of cardiovascular disease. Cuthbert et al.’s 2018 paper demonstrated that patients with pre-existing cardiovascular disease had poorer overall median survival rates, with cardiovascular disease being the most common cause of non-CRC related mortality [20].

A study from Walsh et al. demonstrated poorer all-cause mortality for CRC patients affected by AF [21]. However, this study included patients who were found to be in AF either pre or post-operatively. Post-operative AF has been shown to be a marker of both systemic inflammation and anastomotic breakdown and thus, could account for the high all-cause mortality demonstrated in Walsh’s study [22, 23]. To date, there is no literature that specifically links the relationship between pre-existing atrial fibrillation and post-operative complications following CRC surgery.

COPD was also found to be independently associated with high grade complications. COPD is the second leading cause of disease burden in Australia in patients aged 65–74 [24]. In a 2004 population based study, patients with COPD had a 13-day mortality rate of 13%, compared with only 5.3% without COPD [25]. Lemmens et al. also demonstrated that COPD is associated with higher rates of pneumonia and post-operative bleeding following colorectal cancer resections [13].

One third of all patients with pre-existing atrial fibrillation and 29% of patients with COPD developed high grade post-operative complications. Amongst these patients, there was a higher proportion of medical complications compared to surgical complications. Patients with COPD who developed high grade complications had higher incidences of post-operative respiratory infections, ischaemic cardiac events and had an increased requirement for respiratory and ventilatory support. Patients with AF who developed complications were more likely to develop cardiac arrhythmias and respiratory complications.

Complications arising in those with AF and COPD seem to largely be due to an exacerbation of their underlying cardiorespiratory disease rather than a specific post-operative surgical aetiology. Further to this, atrial fibrillation and COPD also appear to be markers of poorer overall health and a higher comorbid burden in patients.

It is pertinent to note that across all patients within the study, there was a high burden of comorbid disease. Table 1 shows that 307 patients (58%) had an ASA grade of three or above, indicating a severe systemic disease that limits activity [26]. Data from the 2018 Bi-national Colorectal Cancer Audit [2] produced by the Colorectal Surgical Society of Australia and New Zealand (CSSANZ) showed that only 36% of colorectal surgical patients nationally had an ASA of 3 or greater. This indicates that patients in our hospital catchment area undergoing colorectal cancer resections have a higher burden of disease than those nationally which may be a contributing factor to the rates of post-operative complications following complex abdominal surgery.

The association between pre-operative atrial fibrillation and high grade complications following colorectal cancer resection is a novel finding. The association between COPD and poorer surgical outcomes is equally important in order to help optimise patients perioperatively and manage prospective outcomes. Medical optimisation of patients and their comorbidities in the perioperative period is also important in minimising high grade post-operative complications. Collaborating with physicians, anaesthetists, peri-operative medical teams and allied health is crucial in minimising the risk of high grade post-operative complications and improving patient outcomes.

This study highlights the importance of pre-existing atrial fibrillation and COPD as specific diseases which require careful perioperative optimisation, planning and management in order to minimise their impact on the incidence of high grade complications.

## Supporting information

S1 Appendix Clavien-Dindo classifications of post-operative complications. (PDF)

## References

[1] Australian Institute of Health and Welfare. Cancer Data in Australia; Australian Cancer Incidence and Mortality (ACIM): Colon Cancer Canberra: Commonwealth of Australia 2018.

[2] Heriot A, Platell C, Byrne C, Chapuis P, Doudle M, McMurrick P, et al The Bi-National Colorectal Cancer Audit Report. Colorectal Surgical Society of Australia and New Zealand; 2018.

[3] Grosso G, Biondi A, Marventano S, Mistretta A, Calabrese G, Basile F. Major postoperative complications and survival for colon cancer elderly patients. BMC Surg. 2012;12(1):20 10.1186/1471-2482-12-S1-S20 23173563 PMC3499273

[4] Aquina C, Mohile S, Tejani M, Becerra A, Xu Z, Hensley B, et al The impact of age on complications, survival, and cause of death following colon cancer surgery. Br J Cancer. 2017;116:389–97. 10.1038/bjc.2016.421 28056465 PMC5294480

[5] Yap R, Wilkins S, Staples M, Oliva K, McMurrick P. The Effect of Diabetes on the Perioperative Outcomes of Colorectal Cancer Surgery Patients. PLoS One. 2016;11(12):e0167271 10.1371/journal.pone.0167271 27907053 PMC5132159

[6] Tominaga T, Nonaka T, Takeshita H, Kunizaki M, Sumida Y, Hidaka S, et al The Charlson Comorbidity Index as an Independent Prognostic Factor in Older Colorectal Cancer Patients. Indian J Surg. 2018;80(1):54–60. 10.1007/s12262-016-1544-4 29581686 PMC5866797

[7] Pule ML, Buckley E, Niyonsenga T, Roder D. The effects of comorbidity on colorectal cancer mortality in an Australian cancer population. Sci Rep. 2019;9(1):8580 10.1038/s41598-019-44969-8 31189947 PMC6561932

[8] Austin SR, Wong YN, Uzzo RG, Beck JR, Egleston BL. Why Summary Comorbidity Measures Such As the Charlson Comorbidity Index and Elixhauser Score Work. Med Care. 2015;53(9):e65–72. 10.1097/MLR.0b013e318297429c 23703645 PMC3818341

[9] Xu L, Qiu HZ, Wu B, Lin G, Lu J, Zhang G, et al Analysis of Clavien-Dindo classification and its prognosis factors of complications after laparoscopic right hemicolectomy. Chin J Surg. 2018;56(12):900–5. 10.3760/cma.j.issn.0529-5815.2018.12.005 30497116

[10] Duraes L, Stocchi L, Steele S, Kalady M, Church J, Gorgun E, et al The Relationship Between Clavien-Dindo Morbidity Classification and Oncologic Outcomes After Colorectal Cancer Resection. Ann Surg Oncol. 2018;25(1):188–96. 10.1245/s10434-017-6142-6 29116488

[11] Dasgupta P, Youlden DR, Baade PD. An analysis of competing mortality risks among colorectal cancer survivors in Queensland, 1996–2009. Cancer Causes Control. 2013;24(5):897–909. 10.1007/s10552-013-0166-4 23408244

[12] Pedrazzani C, Cerullo G, De Marco G, Marrelli D, Neri A, De Stefano A, et al Impact of age-related comorbidity on results of colorectal cancer surgery. World J Gastroentero. 2009;15(45):5706–11. 10.3748/wjg.15.5706 19960568 PMC2789224

[13] Lemmens VE, Janssen-Heijnen ML, Houterman S, Verheij KD, Martijn H, van de Poll-Franse L, et al Which comorbid conditions predict complications after surgery for colorectal cancer? World J Surg. 2007;31(1):192–9. 10.1007/s00268-005-0711-8 17180570

[14] Ball J, Thompson D, Ski C, Carrington M, Gerber T, Stewart S. Estimating the current and future prevalence of atrial fibrillation in the Australian adult population. Med J Aust. 2015;202(1):32–5. 10.5694/mja14.00238 25588442

[15] Capucci A, Villani G, Aschieri D. Risk of complications of atrial fibrillation. Pacing Clin Electrophysiol. 1997;20(10):2684–91. 10.1111/j.1540-8159.1997.tb06117.x 9358515

[16] Mann C, Kendall S, Lip G. Acute management of atrial fibrillation with acute haemodynamic instability and in the postoperative setting. Heart. 2007;93(1):45–7. 10.1136/hrt.2006.099929 16952970 PMC1861334

[17] Andre C. Preventing bleeding and thromboembolic complications in atrial fibrillation patients undergoing surgery. Arq Neuropsiquiatr. 2015;73(8):704–13. 10.1590/0004-282X20150085 26222364

[18] An Q, Yu T, Cao X, Yang H, Zhao G, Wu G, et al Comparative analysis of postoperative complications on elderly colorectal cancer patients over 65 years with and without comorbid cardiovascular diseases. Chin J Gastrointest Surg. 2016;19(9):1035–39. 27680074

[19] Bokey L, Chapuis P, Keshava A, Rickard MJ, Stewart P, Dent OF. Complications after resection of colorectal cancer in a public hospital and a private hospital. ANZ J Surg. 2015;85(3):128–34. 10.1111/ans.12685 24852703

[20] Cuthbert C, Hemmelgarn B, Xu Y, Cheung W. The effect of comorbidities on outcomes in colorectal cancer survivors: a population-based cohort study. J Cancer Surviv. 2018;12(6):733–43. 10.1007/s11764-018-0710-z 30191524

[21] Walsh SR, Gladwish KM, Ward NJ, Justin TA, Keeling NJ. Atrial fibrillation and survival in colorectal cancer. J Surg Oncol. 2004;29(2):40 10.1186/1477-7819-2-40 15569387 PMC538254

[22] Siu CW, Tung HM, Chu KW, Jim MH, Lau CP, Tse HF. Prevalence and predictors of new-onset atrial fibrillation after elective surgery for colorectal cancer. Pacing Clin Electrophysiol. 2005;28 Suppl 1:S120–3. 10.1111/j.1540-8159.2005.00024.x 15683477

[23] Sutton C, Marshall L, Williams N, Berry D, Thomas W, Kelly M. Colo-rectal anastomotic leakage often masquerades as a cardiac complication. Colorectal Dis. 2004;6(1):21–2. 10.1111/j.1463-1318.2004.00574.x 14692947

[24] Australian Institute of Health and Welfare. Australian Burden of Disease Study 2015: Interactive data on disease burden. Australian Burden of Disease Canberra; 2019.

[25] Platon AM, Erichsen R, Christiansen CF, Andersen LK, Svaerke C, Montomoli J, et al The impact of chronic obstructive pulmonary disease on intensive care unit admission and 30-day mortality in patients undergoing colorectal cancer surgery: a Danish population-based cohort study. BMJ Open Respir Res. 2014;1(1):e000036 10.1136/bmjresp-2014-000036 25478184 PMC4212724

[26] Fitz-Henry J. The ASA classification and peri-operative risk. Ann R Coll Surg Engl. 2011;93(3):185–7. 10.1308/rcsann.2011.93.3.185a 21477427 PMC3348554

