# Supplementary material for: The impact of comorbidities on post-operative complications following colorectal cancer surgery
Source: PLoS One. 2020 Dec 23;15(12):e0243995. doi: 10.1371/journal.pone.0243995 (PMC7757883; doi:10.1371/journal.pone.0243995)
Supplement: S1 Appendix — (PDF) [file pone.0243995.s001.pdf]

Appendix I: Clavien-Dindo classifications of post-operative complications

	Grades	Definition
Low Grade Complications	Grade I	Any deviation from the normal postoperative course without the need for pharmacological treatment or surgical, endoscopic and radiological interventions<br>Allowed therapeutic regimens are: drugs as antiemetic's, antipyretics, analgesics, diuretics and electrolytes and physiotherapy. This grade also includes wound infections opened at the bedside.
	Grade II	Requiring pharmacological treatment with drugs other than such allowed for grade I complications. Blood transfusions and total parenteral nutrition are also included.
High Grade Complications	Grade III	Requiring surgical, endoscopic or radiological intervention
	- IIIa	Intervention not under general anaesthesia
	- IIIb	Intervention under general anaesthesia
	Grade IV	Life-threatening complication (including CNS complications) requiring IC/ICU-management
	- IVa	Single organ dysfunction (including dialysis)
	- IVb	Multi-organ dysfunction
	Grade V	Death of a patient
